# Thymoma in the United States (2000-2023): Population-Based Incidence, Treatment Patterns, and Survival

**DOI:** 10.7759/cureus.109823

**Published:** 2026-05-28

**Authors:** Tahomina Hassan, Mahmudul Hassan

**Affiliations:** 1 Hematology and Oncology, Covenant Health System, Lubbock, USA; 2 Computer Science (Biomedical Informatics), Arizona State University, Tempe, USA

**Keywords:** cancer epidemiology, cox regression, incidence trends, kaplan-meier analysis, population-based study, seer, survival analysis, thoracic oncology, thymoma, treatment patterns

## Abstract

Purpose: Thymoma is a rare anterior mediastinal tumor for which contemporary US population-based data remain limited. The primary objectives of this study were to estimate age-adjusted thymoma incidence from 2000 to 2023 and to identify independent predictors of overall survival using multivariable Cox regression. Secondary objectives included characterizing demographic distributions, treatment patterns, and subgroup survival differences in a contemporary national cohort.

Methods: We conducted a retrospective population-based study using the Surveillance, Epidemiology, and End Results (SEER) 17 Registries Research Data, November 2025 submission, including patients diagnosed with thymoma between 2000 and 2023. Cases were identified using primary site C37.9 and ICD-O-3 histology codes 8580-8585. Age-adjusted incidence rates were calculated per 100,000 population. Overall survival was assessed with Kaplan-Meier methods, and multivariable Cox proportional hazards regression was used to identify factors associated with mortality.

Results: The SEER incidence dataset included 5,706 thymoma cases, of which 5,697 had corresponding case-level data for descriptive analyses. The median age at diagnosis was 61 years, and 47.9% of patients were 50-69 years old. The overall age-adjusted incidence rate was 0.271 per 100,000 population. Incidence remained relatively stable through 2020, then increased to 0.570 in 2021 and 0.674 in 2023. Rates were slightly higher in men than in women (0.285 versus 0.261 per 100,000) and highest among Asian or Pacific Islander patients (0.473 per 100,000). Surgery was the most common treatment and was independently associated with better survival (hazard ratio (HR): 0.34, 95% confidence interval (CI): 0.29-0.40). In contrast, age 70 years or older, tumor size greater than 5 cm, and type B3 histology were associated with increased mortality.

Conclusions: In this contemporary SEER-based analysis, thymoma incidence remained low and largely stable from 2000 through 2020; reported incidence increased after 2020, although this pattern may reflect changes in registry case ascertainment rather than a true epidemiologic shift. Surgery was the strongest modifiable predictor of survival, whereas older age, larger tumor size, and type B3 histology were associated with worse outcomes. Radiation therapy was not independently associated with survival in the adjusted model, but this finding reflects confounding by indication inherent to the observational study design and should not be interpreted as evidence against radiotherapy efficacy.

## Introduction

Thymoma is the most common primary tumor of the anterior mediastinum, yet it remains a rare malignancy at the population level [1,2]. Its rarity has limited the feasibility of large prospective studies and randomized trials, so much of the available evidence has come from retrospective institutional series and population-based registry analyses [1,3-6]. Although thymoma is often regarded as a relatively indolent tumor, its clinical course is heterogeneous. Some patients experience prolonged survival after complete resection, whereas others present with more invasive disease and less favorable outcomes, highlighting the need for contemporary population-level data that can better define its epidemiology and prognosis [2,3].

Large cancer registries have therefore played a central role in improving our understanding of thymoma in the United States. Earlier studies using the Surveillance, Epidemiology, and End Results (SEER) program showed that thymoma occurs predominantly in middle-aged and older adults and that incidence varies across racial and ethnic groups, with consistently higher rates reported among Asian or Pacific Islander populations [1,4,5]. Registry-based work has also suggested that patients with thymoma may have an increased risk of second primary malignancies, particularly hematologic cancers, which adds further clinical importance to long-term follow-up in this population [7]. Taken together, these studies have established a useful epidemiologic foundation, but many were based on earlier registry periods and did not fully reflect more recent data or current patterns of care [1,4,5,7].

In addition to incidence patterns, prior national analyses have shown that prognosis is driven mainly by disease extent at diagnosis and the feasibility of surgical resection [2,3,8]. Surgery remains the cornerstone of treatment for patients with localized or potentially resectable disease, while age, histologic subtype, and tumor size provide additional prognostic information [2,3,8,9]. At the same time, treatment-related findings from registry studies must be interpreted cautiously. Variables for radiation and systemic therapy are often limited to first-course treatment, clinical details on recurrence and resection margins are usually unavailable, and observational comparisons remain vulnerable to confounding by indication [6,10]. These limitations do not diminish the value of registry studies in rare diseases such as thymoma, but they do shape how treatment associations should be understood and applied in clinical practice [6,10].

Despite the value of earlier registry work, contemporary US population-based data that integrate incidence trends, demographic patterns, treatment approaches, and survival outcomes in a single analysis remain limited. This is particularly relevant because registry coverage, coding practices, and the available duration of follow-up have continued to evolve. An updated analysis using the most recent available SEER release can therefore provide a more current picture of thymoma in the United States and help place recent survival and treatment patterns in the context of prior national evidence [4-6].

In this study, we used the SEER 17 Registries Research Data (November 2025 submission) to examine thymoma in the United States from 2000 to 2023. The primary objectives were to estimate age-adjusted incidence trends over the study period and identify independent predictors of overall survival using multivariable Cox proportional hazards regression. Secondary objectives were to characterize demographic distributions, treatment patterns, and subgroup-specific survival differences in a contemporary national cohort. We hypothesized that surgical resection would remain the strongest modifiable determinant of survival, while older age and larger tumor size would be associated with inferior outcomes.

## Materials and methods

Study design and data source

We conducted a retrospective population-based cohort study using the Surveillance, Epidemiology, and End Results (SEER) program of the National Cancer Institute. SEER provides standardized population-level data on cancer incidence, patient demographics, tumor characteristics, first-course treatment, and survival, and is widely used for epidemiologic research in rare malignancies [6,10,11]. For this study, we used the SEER 17 Registries Research Data, November 2025 submission, and included patients diagnosed between January 1, 2000, and December 31, 2023 [11,12].

For incidence analyses, age-adjusted rates were generated in SEER*Stat from the same SEER 17 database. Overall incidence and sex-stratified and race/ethnicity-stratified estimates were derived for the 2000-2023 period. Using a single registry source for both incidence and case-level analyses ensured consistency across all study components [11,12].

Case ascertainment and study population

Patients with thymoma were identified using primary site code C37.9 (thymus) and ICD-O-3 histology codes 8580-8585. Histologic subtypes were classified as type A (8581), type AB (8582), type B1 (8583), type B2 (8584), and type B3 (8585). Cases reported by autopsy only or death certificate only were excluded from survival analyses to improve the reliability of outcome estimates [11].

Variables

Demographic variables included age at diagnosis, sex, and race/ethnicity. Race and ethnicity were categorized as White, Black, Asian or Pacific Islander, and Hispanic, consistent with prior SEER-based epidemiologic studies of thymoma and other cancers [1,4,5]. In this study, White, Black, and Asian or Pacific Islander refer to non-Hispanic categories derived from the SEER race and origin recode, whereas Hispanic includes all races.

Tumor-related variables included histologic subtype and tumor size. Tumor size was derived from the SEER “Tumor Size Over Time Recode” variable and categorized as ≤5 cm or >5 cm, a threshold that has been used in prior registry-based prognostic studies of thymic malignancies [9]. Treatment variables included surgery and radiation therapy, as recorded in the first-course treatment fields. Surgical treatment was defined using SEER site-specific surgery codes, including harmonized 2023 A-codes, whereas radiation therapy was based on the SEER “Radiation Recode” variable and classified as yes versus no/unknown [11].

Outcomes

The primary outcome was overall survival, defined as the interval from diagnosis to death from any cause or last follow-up. Survival time was measured using the SEER “Survival months” variable, and censoring status was determined from the “Vital Status Recode” variable. Patients who were alive at the last follow-up were censored. Secondary analyses examined survival according to age group, treatment status, histologic subtype, and tumor size.

Statistical analysis

Baseline characteristics were summarized using descriptive statistics, with categorical variables reported as frequencies and percentages. Age-adjusted incidence rates were calculated per 100,000 population using SEER*Stat software (version 9.0.43) and standardized to the 2000 US standard population [11,12]. Incidence rate sessions were exported directly from SEER*Stat using the SEER 17 Registries Research Data, November 2025 submission (2000-2023), with age adjustment performed internally by the software. Overall survival was estimated with the Kaplan-Meier method and compared across groups using the log-rank test. Multivariable Cox proportional hazards regression was used to identify independent predictors of mortality. Covariates were selected a priori based on clinical relevance and prior literature and included age group (≥70 versus <70 years), sex (male versus female), race/ethnicity (non-Hispanic Black, non-Hispanic Asian or Pacific Islander, and Hispanic versus non-Hispanic White as reference), histologic subtype (AB, B1, B2, B3 versus type A as reference), tumor size (>5 cm versus ≤5 cm), surgery (received versus not received), and radiation therapy (received versus not received). The proportional hazards assumption was assessed by visual inspection of log-log survival plots and Schoenfeld residual tests; no significant violations were identified. Hazard ratios (HRs) and 95% confidence intervals (CIs) were reported, and a two-sided p-value of <0.05 was considered statistically significant. Case-level analyses, Kaplan-Meier estimation, and Cox regression were performed using Python (version 3.12.9; Python Software Foundation, Wilmington, DE) with the lifelines package (version 0.30.3) and pandas (version 2.3.3) [10-12]. This study is reported in accordance with the Strengthening the Reporting of Observational Studies in Epidemiology (STROBE) guidelines for cohort studies [13].

Ethics statement

This study used deidentified data from the Surveillance, Epidemiology, and End Results (SEER) program of the National Cancer Institute. The database contains registry-based cancer information without direct patient identifiers and is available to researchers through a data use agreement. Because this was a secondary analysis of deidentified data with no direct patient contact, institutional review board approval and informed consent were not required. All analyses were conducted in accordance with SEER data use requirements.

## Results

Patient characteristics

A total of 5,706 patients with thymoma diagnosed between 2000 and 2023 were identified in the SEER incidence dataset, of whom 5,697 were available for case-level descriptive analyses. The median age at diagnosis was 61 years, and nearly half of the patients (47.9%) were aged 50-69 years. Thymoma occurred in both sexes with a nearly even distribution, with a slight predominance of women (50.5%). Most patients were White (54.9%), followed by Asian or Pacific Islander (17.8%) and Black individuals (14.4%), while Hispanic patients accounted for 11.6% of the cohort. Baseline demographic, clinicopathologic, and treatment characteristics are summarized in Table 1.

**Table 1:** Baseline characteristics of patients with thymoma in SEER (2000-2023) SEER: Surveillance, Epidemiology, and End Results, NOS: not otherwise specified Note: Percentages are calculated within the displayed categories. Some category totals may not sum to 100% because omitted groups in the manuscript-facing table include non-Hispanic American Indian/Alaska Native, non-Hispanic unknown race, histology NOS/other, and unknown tumor size. A total of 5,706 thymoma cases were identified in the SEER incidence dataset, of which 5,697 were available in the corresponding case-level extract for descriptive analyses. White, Black, and Asian or Pacific Islander denote non-Hispanic categories based on the SEER race and origin recode; Hispanic includes all races.

Characteristic	Number (%)
Total patients	5,697
Age group	
<50 years	1,379 (24.2%)
50-69 years	2,728 (47.9%)
≥70 years	1,590 (27.9%)
Sex	
Male	2,818 (49.5%)
Female	2,879 (50.5%)
Race/ethnicity	
White	3,129 (54.9%)
Black	823 (14.4%)
Asian or Pacific Islander	1,013 (17.8%)
Hispanic	662 (11.6%)
Treatment	
Surgery	4,465 (78.4%)
Radiation	2,019 (35.4%)
Tumor size	
≤5 cm	1,835 (32.2%)
>5 cm	2,977 (52.3%)
Histology	
Type A	539 (9.5%)
Type AB	1,265 (22.2%)
Type B1	726 (12.7%)
Type B2	952 (16.7%)
Type B3	745 (13.1%)

Among histologic subtypes, type AB thymoma was the most common (22.2%), followed by type B2 (16.7%) and type B3 (13.1%). Tumors measuring >5 cm were more frequent than those ≤5 cm (52.3% versus 32.2% among cases with recorded tumor size). Surgery was the most frequently recorded treatment (78.4%), whereas radiation therapy was used in 35.4% of patients. Overall, these findings indicate that thymoma in the United States during the study period was diagnosed mainly in middle-aged and older adults and was most often managed surgically.

Incidence trends (2000-2023)

**Figure 1:**
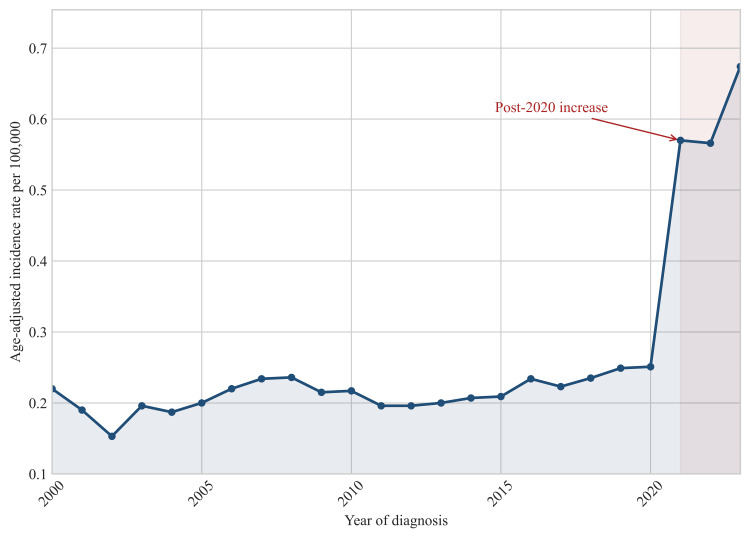
Annual age-adjusted incidence of thymoma in the United States (2000-2023) SEER: Surveillance, Epidemiology, and End Results Rates were derived from SEER*Stat rate-session exports using the SEER 17 Registries Research Data, November 2025 submission, and were age-adjusted to the 2000 US standard population. Incidence remained relatively stable from 2000 through 2020 (0.220-0.251 per 100,000 population) before increasing to 0.570 in 2021, 0.566 in 2022, and 0.674 in 2023.

The overall age-adjusted incidence rate of thymoma during the study period was 0.271 per 100,000 population. Annual incidence remained relatively stable from 2000 through 2020, with rates fluctuating within a narrow range, then increased during 2021-2023. The annual rate rose from 0.220 per 100,000 in 2000 to 0.249 in 2019 and 0.251 in 2020, followed by higher rates of 0.570 in 2021, 0.566 in 2022, and 0.674 in 2023. These annual estimates are presented in Table 2, and the temporal pattern is illustrated in Figure 1.

**Table 2:** Annual incidence rates of thymoma in the United States (2000-2023) SEER: Surveillance, Epidemiology, and End Results Note: Rates were derived from SEER*Stat rate-session exports using the SEER 17 Registries Research Data, November 2025 submission (2000-2023). Rates are age-adjusted to the 2000 US standard population. Selected calendar years are shown to preserve a compact layout while highlighting the recent increase in incidence.

Year	Cases	Age-adjusted rate (per 100,000)
2000	157	0.220
2005	155	0.200
2010	183	0.217
2015	201	0.209
2019	245	0.249
2020	257	0.251
2021	574	0.570
2022	582	0.566
2023	708	0.674

Although the increase in the final years of the study period was notable, the overall pattern across two decades was one of low and largely stable incidence. Given the rarity of thymoma, these recent changes should be interpreted cautiously, as even modest shifts in case reporting or ascertainment can influence annual age-adjusted rates.

Incidence by sex

Sex-stratified incidence analyses showed a slightly higher age-adjusted incidence rate in men than in women. The rate was 0.285 per 100,000 in men compared with 0.261 per 100,000 in women, despite the similar overall case distribution between sexes. These findings are summarized in Table 3.

**Table 3:** Incidence of thymoma by sex (2000-2023) SEER: Surveillance, Epidemiology, and End Results Note: Values were derived from SEER*Stat sex-stratified rate-session exports using the SEER 17 Registries Research Data, November 2025 submission (2000-2023). Rates are age-adjusted to the 2000 US standard population. Sex-stratified incidence showed a modest male predominance over the study period.

Sex	Cases	Age-adjusted rate (per 100,000)
Male	2,820	0.285
Female	2,886	0.261

Incidence by race and ethnicity

Incidence varied across racial and ethnic groups. The highest age-adjusted rate was observed among Asian or Pacific Islander patients (0.473 per 100,000), followed by Black patients (0.404 per 100,000). Lower rates were seen among White (0.233 per 100,000) and Hispanic (0.210 per 100,000) populations. These data are presented in Table 4 and indicate a clear demographic gradient in thymoma incidence across the US population.

**Table 4:** Incidence of thymoma by race/ethnicity (2000-2023) SEER: Surveillance, Epidemiology, and End Results Note: Values were derived from SEER*Stat race/ethnicity-stratified rate-session exports using the SEER 17 Registries Research Data, November 2025 submission (2000-2023). Rates are age-adjusted to the 2000 US standard population. The highest incidence was observed among Asian or Pacific Islander patients, followed by Black patients. White, Black, and Asian or Pacific Islander denote non-Hispanic categories based on the SEER race and origin recode; Hispanic includes all races.

Race/ethnicity	Cases	Age-adjusted rate (per 100,000)
White	3,135	0.233
Black	824	0.404
Asian or Pacific Islander	1,014	0.473
Hispanic	663	0.210

Treatment patterns

Surgery was the predominant treatment modality in this cohort. Surgery alone was the most common treatment category, accounting for 48.7% of patients, followed by combined surgery and radiation therapy in 29.6%. Radiation alone was used in 5.8%, whereas 15.8% of patients had no treatment or unknown treatment recorded in the registry. These distributions are shown in Table 5.

**Table 5:** Treatment patterns among patients with thymoma SEER: Surveillance, Epidemiology, and End Results, NOS: not otherwise specified Note: Values were derived from the case-level thymoma cohort. Surgery was defined using site-specific surgery codes from 1998 to 2022 and harmonized 2023 A-codes. Radiation was defined as administered beam radiation, radiation NOS, radioisotopes, or combination radiation; refused or recommended-but-unknown radiation was not counted as administered therapy.

Treatment category	Number (%)
Surgery only	2,777 (48.7%)
Radiation only	331 (5.8%)
Surgery + radiation	1,688 (29.6%)
No treatment/unknown	901 (15.8%)

Overall, the treatment pattern suggests that resection remained the central management approach for thymoma during the study period, while radiation therapy was used more selectively, often in combination with surgery rather than as a sole treatment.

Survival analysis

Overall survival was assessed using Kaplan-Meier methods to describe survival patterns across key clinical and treatment-related subgroups. Survival was examined according to surgical treatment, age group, radiation therapy, histologic subtype, and tumor size. These analyses were descriptive and were intended to provide a population-level overview of prognostic differences among patients with thymoma. Kaplan-Meier estimates of median overall survival, 5-year overall survival, and 10-year overall survival by subgroup are summarized in Table 6.

**Table 6:** Summary survival estimates by subgroup OS: overall survival, mo: months, NH: non-Hispanic, PI: Pacific Islander, NOS: not otherwise specified Note: Overall survival estimates were derived using the Kaplan-Meier method. This table summarizes median overall survival, 5-year overall survival, and 10-year overall survival for the full cohort and clinically relevant subgroups, including histologic subtype, tumor size, age group, sex, race/ethnicity, surgery, and radiation therapy. The table is provided to support the survival estimates reported in the Results section and improve readability by presenting subgroup survival outcomes in one location.

Subgroup	Number	Events	Median OS (mo)	5-year OS	10-year OS
Overall	5,697	1,873	151	76.5%	58.2%
Histologic subtype					
Type A	539	162	148	76.2%	59.3%
Type AB	1,265	245	178	85.4%	64.7%
Type B1	726	198	174	79.7%	65.6%
Type B2	952	238	169	80.0%	60.5%
Type B3	745	304	152	72.3%	54.9%
NOS	1,470	726	125	69.4%	50.8%
Tumor size					
≤5 cm	1,835	409	186	84.0%	68.0%
>5 cm	2,977	1,001	148	76.8%	57.9%
Unknown	885	463	101	62.7%	44.0%
Age					
<70 years	4,107	1,131	201	82.9%	66.5%
≥70 years	1,590	742	78	58.5%	34.1%
Sex					
Male	2,818	1,009	144	73.9%	56.7%
Female	2,879	864	159	79.1%	59.6%
Race/ethnicity					
NH White	3,129	1,108	151	76.4%	58.4%
NH Black	823	285	133	75.7%	53.0%
NH Asian/PI	1,013	287	169	79.1%	60.4%
Hispanic	662	183	156	73.5%	59.5%
Surgery					
Surgery received	4,465	1,173	183	84.2%	66.3%
No surgery	1,232	700	60	49.4%	30.6%
Radiation					
Radiation received	2,019	768	159	78.9%	60.8%
No radiation	3,678	1,105	143	75.1%	56.4%

Survival by surgery

**Figure 2:**
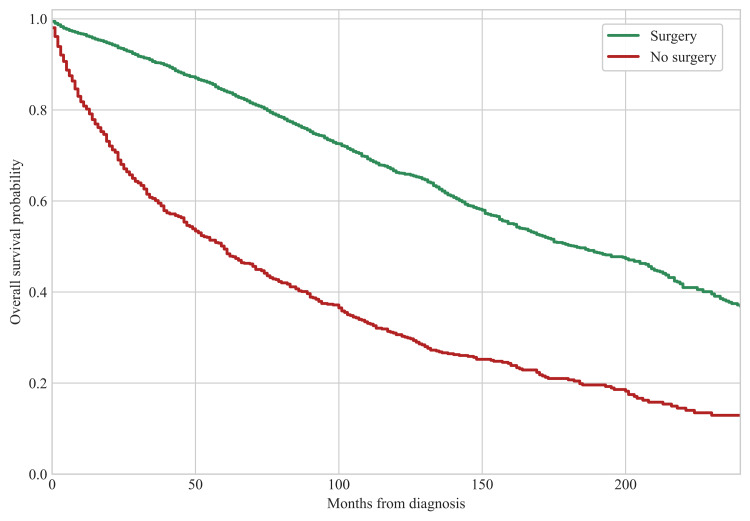
Kaplan-Meier overall survival curves according to surgical treatment in patients with thymoma Overall survival was significantly better among patients who underwent surgical resection compared with those who did not undergo surgery.

Kaplan-Meier analyses showed clear differences in overall survival according to surgical treatment. Patients who underwent surgery had substantially better survival than those who did not, and this separation persisted throughout follow-up. The survival curves according to surgical treatment are shown in Figure 2.

Survival by age group

**Figure 3:**
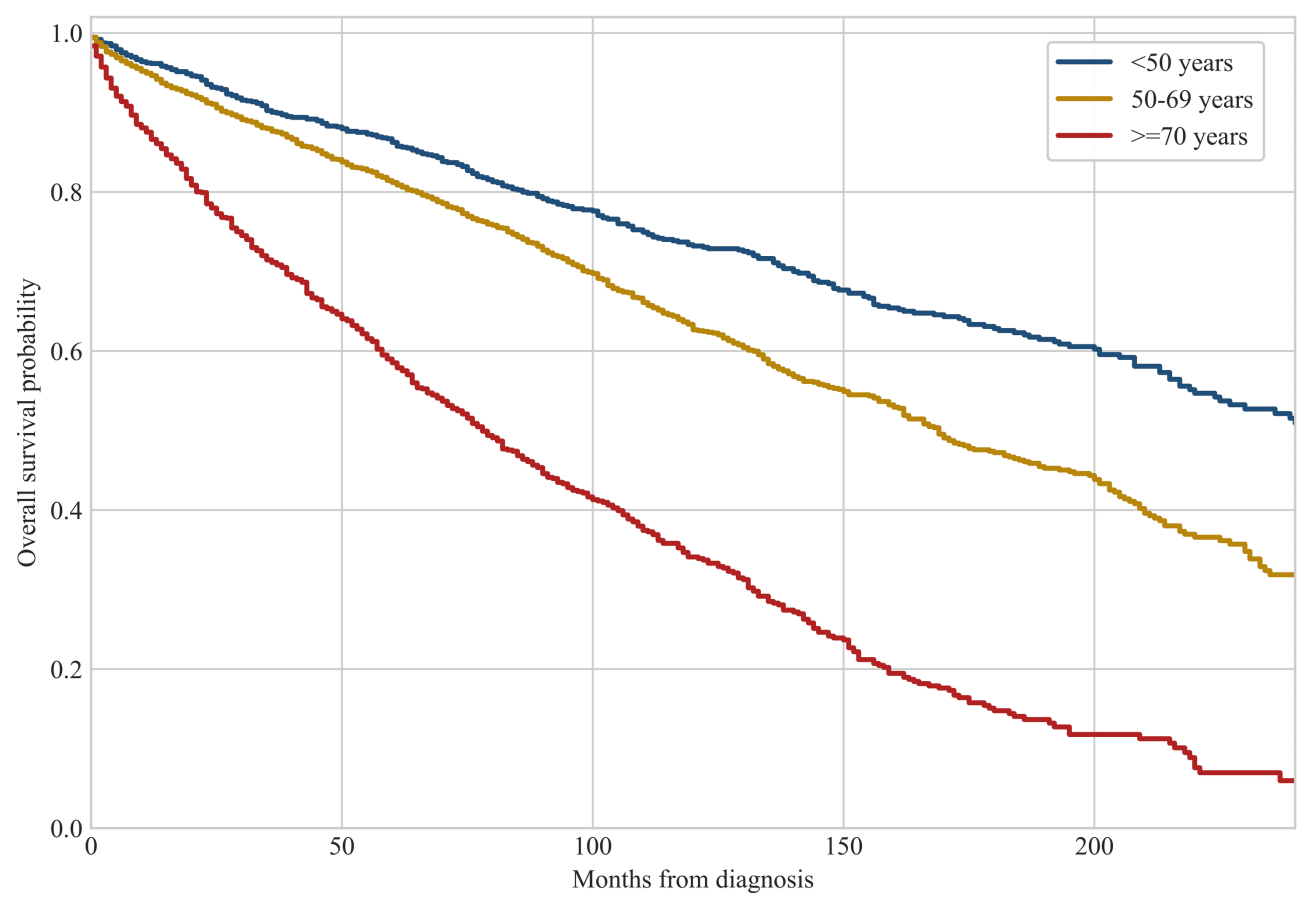
Kaplan-Meier overall survival curves according to age group in patients with thymoma Overall survival was compared across patients aged <50 years, 50-69 years, and ≥70 years.

Overall survival also differed markedly by age group. Patients aged ≥70 years had substantially worse survival than younger patients, whereas survival was more favorable among those diagnosed before age 70. Kaplan-Meier survival curves by age group are presented in Figure 3.

Survival by radiation therapy

Radiation therapy showed a modestly favorable association with overall survival in unadjusted analyses. Kaplan-Meier estimates of 5-year overall survival were 78.9% in patients who received radiation and 75.2% in those who did not, with corresponding 10-year rates of 60.8% and 56.6%, respectively. The unadjusted median overall survival was 159 months with radiation versus 143 months without radiation (log-rank p<0.001). However, these unadjusted findings should not be interpreted as evidence of radiotherapy benefit. In the adjusted model, radiation therapy was not independently associated with overall survival (HR: 0.94, 95% CI: 0.82-1.07; p=0.362; Table 7). This adjusted result should also not be interpreted as evidence for or against radiotherapy efficacy, because patients receiving radiation in SEER were likely to have higher-risk disease features, such as more advanced stage, unresectable disease, or incomplete resection, which are not fully captured in the dataset. Therefore, the radiation findings are best understood as observational associations affected by confounding by indication rather than clinical treatment-effect estimates [6,9-12].

**Table 7:** Multivariable Cox proportional hazards analysis of factors associated with overall survival HR: hazard ratio, CI: confidence interval, SEER: Surveillance, Epidemiology, and End Results Note: Estimates were generated from a multivariable Cox proportional hazards model for overall survival using a complete-case subset with known tumor size and displayed race/histology categories (N=3,714). Reference categories were age <70 years, female sex, White race, histology type A, tumor size ≤5 cm, no surgery, and no radiation. White and Black denote non-Hispanic categories based on the SEER race and origin recode.

Covariate	Comparison	HR	95% CI	p-value
Age	≥70 years versus <70 years	3.08	2.68-3.55	<0.001
Sex	Male versus female	1.22	1.07-1.39	0.003
Race/ethnicity	Black versus White	1.12	0.92-1.37	0.253
Histology	Type B3 versus type A	1.31	1.06-1.62	0.014
Tumor size	>5 cm versus ≤5 cm	1.40	1.21-1.61	<0.001
Surgery	Yes versus no	0.34	0.29-0.40	<0.001
Radiation	Yes versus no	0.94	0.82-1.07	0.362

Subgroup analysis

Subgroup analyses were conducted to better characterize survival patterns across clinically relevant tumor features. We focused on histologic subtype and tumor size because these factors are commonly used in thymoma risk assessment and were available in the SEER dataset. These analyses provided additional context for the Kaplan-Meier findings and helped support the interpretation of the multivariable survival model.

Histologic subtype

Survival differed significantly across histologic subtypes in unadjusted analyses (log-rank p<0.001). Five-year overall survival was highest for type AB thymoma (85.4%), followed by type B2 (80.0%), type B1 (79.7%), type A (76.2%), and type B3 (72.3%); patients classified as thymoma not otherwise specified (NOS) had the lowest five-year rate (69.4%). Ten-year overall survival followed a similar pattern: type B1, 65.6%; type AB, 64.7%; type B2, 60.5%; type A, 59.3%; type B3, 54.9%; and NOS, 50.8%. Kaplan-Meier median overall survival ranged from 178 months (type AB) to 125 months (NOS). Patients with type A and type AB thymoma had more favorable outcomes, whereas those with type B3 thymoma had poorer survival. This pattern was consistent with the adjusted analysis, in which type B3 histology, compared with type A, was associated with increased mortality (HR: 1.31, 95% CI: 1.06-1.62; p=0.014; Table 7).

Tumor size

Tumor size was also significantly associated with prognosis in unadjusted analyses (log-rank p<0.001). Five-year overall survival was 84.0% for tumors ≤5 cm and 76.8% for tumors >5 cm, with corresponding 10-year rates of 68.0% and 57.9%, respectively. Kaplan-Meier median overall survival was 186 months for tumors ≤5 cm versus 148 months for tumors >5 cm. This was consistent with the multivariable model, where tumor size >5 cm remained independently associated with poorer survival (HR: 1.40, 95% CI: 1.21-1.61; p<0.001; Table 7).

Multivariable analysis

In multivariable Cox proportional hazards regression, several factors remained independently associated with overall survival (Table 7). The Cox model included 3,714 patients with complete data for the modeled covariates. Patients included in and excluded from the complete-case Cox model were compared descriptively in Table 8 to describe the missing data structure and assess potential selection bias. After adjustment for demographic, tumor-related, and treatment variables, older age, male sex, larger tumor size, and type B3 histology were associated with increased mortality.

**Table 8:** Comparison of patients included versus excluded from the Cox proportional hazards model IQR: interquartile range, NOS: not otherwise specified, WHO: World Health Organization Note: This table compares patients included in the complete-case Cox proportional hazards model with those excluded because of unavailable modeled covariates, primarily thymoma NOS classification, unknown tumor size, or small race/ethnicity categories. p-values were calculated using the Mann-Whitney U test for continuous variables and chi-square tests for categorical variables. The comparison is provided to describe the missing data structure and assess potential selection bias, directly addressing the reduction from the full case-level cohort to the Cox model subset. Multiple imputation was not performed because the primary source of exclusion was a structural lack of WHO histologic subtype information rather than randomly missing values.

Variable	Included (n = 3,714)	Excluded (n = 1,983)	p-value
Demographics			
Age, median (IQR)	62 (51-71)	61 (49-71)	0.212
Age ≥70 years	1,020 (27.5%)	570 (28.7%)	0.305
Male sex	1,826 (49.2%)	992 (50.0%)	0.536
Race/ethnicity			
Non-Hispanic White	2,102 (56.6%)	1,027 (51.8%)	<0.001
Non-Hispanic Black	502 (13.5%)	321 (16.2%)	0.006
Non-Hispanic Asian/Pacific Islander	674 (18.1%)	339 (17.1%)	0.322
Hispanic	436 (11.7%)	226 (11.4%)	0.701
Other/unknown	0 (0.0%)	70 (3.5%)	<0.001
Histologic subtype			
Type A	471 (12.7%)	68 (3.4%)	<0.001
Type AB	1,130 (30.4%)	135 (6.8%)	<0.001
Type B1	633 (17.0%)	93 (4.7%)	<0.001
Type B2	842 (22.7%)	110 (5.5%)	<0.001
Type B3	638 (17.2%)	107 (5.4%)	<0.001
NOS	0 (0.0%)	1,470 (74.1%)	<0.001
Tumor size			
Tumor size known	3,714 (100.0%)	1,098 (55.4%)	<0.001
Tumor size >5 cm (if known)	2,272 (61.2%)	705 (64.2%)	0.069
Treatment			
Surgery received	3,340 (89.9%)	1,125 (56.7%)	<0.001
Radiation received	1,317 (35.5%)	702 (35.4%)	0.964
Outcomes			
Deceased	945 (25.4%)	928 (46.8%)	<0.001
Survival months, median (IQR)	40 (15-100)	55 (17-128)	<0.001
Diagnosis era			
Diagnosis year, median (IQR)	2018 (2011-2022)	2012 (2005-2020)	<0.001
2000-2009	769 (20.7%)	806 (40.6%)	<0.001
2010-2019	1,333 (35.9%)	669 (33.7%)	0.105
2020-2023	1,612 (43.4%)	508 (25.6%)	<0.001

Age was the strongest predictor of mortality. Compared with patients aged <70 years, those aged ≥70 years had more than a threefold higher risk of death (HR: 3.08, 95% CI: 2.68-3.55; p<0.001). Male sex was also associated with worse survival (HR: 1.22, 95% CI: 1.07-1.39; p=0.003).

Tumor-related characteristics were likewise prognostically important. Tumor size >5 cm was independently associated with poorer survival (HR: 1.40, 95% CI: 1.21-1.61; p<0.001), and type B3 histology, compared with type A, was associated with increased mortality (HR: 1.31, 95% CI: 1.06-1.62; p=0.014). In contrast, Black race, compared with White race, was not significantly associated with overall survival after adjustment (HR: 1.12, 95% CI: 0.92-1.37; p=0.253).

Treatment effects were most pronounced for surgery. Patients who underwent surgery had a markedly lower risk of death than those who did not (HR: 0.34, 95% CI: 0.29-0.40; p<0.001). Radiation therapy was not independently associated with survival in the adjusted model (HR: 0.94, 95% CI: 0.82-1.07; p=0.362). Sensitivity analyses excluding 2023 cases to address the A-code coding transition and excluding 2021-2023 cases to assess the influence of the post-2020 ascertainment period yielded hazard ratios that were generally consistent in direction, magnitude, and statistical significance across covariates, suggesting that these coding and ascertainment issues did not materially affect the primary model findings.

Overall, the multivariable findings summarized in Table 7 indicate that older age, larger tumors, and more aggressive histology were associated with worse outcomes, whereas surgical treatment was strongly associated with improved survival in this national cohort.

## Discussion

This population-based analysis provides an updated overview of thymoma in the United States across more than two decades. In this contemporary SEER cohort, thymoma remained a rare malignancy with a low overall incidence, a predominance in middle-aged and older adults, and clear demographic differences across racial and ethnic groups. Surgery was the most frequently recorded treatment and was strongly associated with improved overall survival, whereas older age, larger tumor size, and more aggressive histologic subtype were associated with worse outcomes. Together, these findings extend earlier US registry-based observations and provide a current national perspective on thymoma incidence, treatment patterns, and prognosis [1-5].

The incidence estimate in the present study confirms the rarity of thymoma while providing an updated benchmark based on the current SEER 17 Registries release through 2023. In our dataset, the overall age-adjusted incidence rate for 2000-2023 was 0.271 per 100,000 population. Annual incidence remained relatively stable from 2000 through 2020, generally ranging from 0.153 to 0.251 per 100,000, before increasing to 0.570 in 2021, 0.566 in 2022, and 0.674 in 2023. The most notable incidence finding was the sharp increase after 2020, particularly from 0.251 per 100,000 in 2020 to 0.570 in 2021. Several observations suggest that this pattern may reflect improved case ascertainment in the SEER November 2025 submission rather than a true biologic increase in thymoma incidence. First, population denominators remained stable across this period, arguing against registry-coverage expansion as the primary explanation. Second, the ICD-O-3 morphology codes used to define thymoma were unchanged, making histologic reclassification less likely. Third, the increase was accompanied by a marked rise in laboratory-only reported cases, suggesting enhanced pathology-based case capture in recent submission years. Because thymoma is rare, even modest changes in ascertainment can substantially influence annual age-adjusted rates. Therefore, the post-2020 increase should be interpreted as a reported incidence increase rather than definitive evidence of a true epidemiologic shift. Comparison with long-standing SEER 8/9 registries was not feasible for the 2021-2023 period and should be pursued in future studies to determine whether this pattern persists in stable registry subsets [4-6,10-12].

The demographic patterns in our cohort also align with prior US registry studies. Thymoma occurred predominantly in middle-aged and older adults, and incidence was highest among Asian or Pacific Islander patients, followed by Black patients [1,4,5]. These differences have been observed repeatedly across prior registry-based analyses and remain one of the most reproducible epidemiologic features of thymoma in the United States [1,4,5]. Although the mechanisms underlying these disparities are not fully understood, their persistence across registry periods suggests that biologic susceptibility, environmental exposures, access to care, and diagnostic practices may all contribute. Our findings therefore reinforce the importance of considering population heterogeneity when interpreting national thymoma incidence patterns [1,4,5].

With respect to treatment, surgery remained the dominant management strategy in this cohort and showed the strongest association with improved survival. This finding is consistent with the current clinical understanding that complete resection remains the cornerstone of treatment for resectable thymoma and an important determinant of long-term outcome [2,3,8]. Although registry data cannot capture important operative details such as completeness of resection, margin status, or surgical approach, the magnitude of the association observed in our multivariable model supports the central role of surgery in routine care. This association should still be interpreted in the context of treatment selection, because patients who undergo surgery are more likely to have resectable disease and more favorable baseline prognostic features than those managed non-operatively [2,3,8].

Radiation therapy was not independently associated with overall survival after multivariable adjustment. This finding should not be interpreted as evidence that radiotherapy lacks clinical value in thymoma. In SEER and other observational registry datasets, radiation is not assigned randomly; it is often used in patients with more advanced disease, incomplete resection, unresectable tumors, or other high-risk features that are incompletely captured in registry variables. This creates substantial confounding by indication and limits any causal interpretation of the radiation-survival association [6,10-12]. Prior SEER-based propensity-matched research reported a potential survival benefit from postoperative radiotherapy in selected higher-risk patients, particularly those with advanced-stage thymoma, underscoring that the present analysis should not be used to judge radiotherapy efficacy [9]. Rather, the non-significant radiation HR in our model most likely reflects the limitations of registry-based treatment-effect inference in the absence of detailed Masaoka-Koga stage, margin status, recurrence, and treatment-intent data [6,9-12].

Several clinicopathologic factors were independently associated with prognosis. Older age at diagnosis was the strongest adverse predictor of overall survival, which is consistent with prior population-based studies and likely reflects both tumor-related risk and competing mortality [3]. Tumor size was also associated with outcome. In Kaplan-Meier analysis, the median overall survival was 186 months for tumors 5 cm or smaller compared with 148 months for tumors larger than 5 cm, and tumor size greater than 5 cm remained independently associated with poorer survival in the multivariable model. These findings are in line with recent SEER-based work suggesting that tumor size provides clinically relevant prognostic information in thymic malignancies [13]. Histologic subtype further contributed to prognostic stratification: type B3 thymoma was associated with poorer survival than type A disease, and unadjusted survival patterns also suggested less favorable outcomes for type B2 and B3 tumors overall. Although registry-based pathology data cannot fully capture the biologic complexity of thymic epithelial tumors, these findings remain consistent with the established clinical impression that more aggressive histologic subtypes carry a worse prognosis [2,13].

This study has several strengths. It uses a large, population-based national dataset and includes the most recent currently available SEER release, allowing contemporary assessment of incidence, treatment, and survival over a long observation period. This is particularly important for a rare tumor such as thymoma, for which single-center studies are often limited by small sample size and restricted generalizability [1,4,6]. By evaluating incidence patterns, treatment utilization, Kaplan-Meier survival, and multivariable prognostic factors within the same study framework, the analysis provides an integrated population-level view of thymoma in the United States.

Several limitations should be acknowledged. Most importantly, SEER does not include Masaoka-Koga stage, which is the principal clinical staging system for thymoma and an important determinant of recurrence and survival [2,3,8]. As a result, the multivariable Cox model could not directly adjust for disease extent, and the reported hazard ratios, particularly those for surgery and tumor size, should be interpreted as prognostic associations rather than stage-independent effects. These findings should not be used for clinical risk stratification without recognizing stage as an important unmeasured confounder. A second important limitation relates to the abrupt increase in age-adjusted incidence after 2020, from 0.251 per 100,000 in 2020 to 0.570 in 2021. This pattern affects the interpretation of incidence trends and should be viewed cautiously. As noted above, stable population denominators, unchanged ICD-O-3 histology codes, and a substantial increase in laboratory-only reported cases suggest that the post-2020 rise may reflect improved case ascertainment in the November 2025 SEER submission rather than a true biologic increase in thymoma incidence. Until this pattern is confirmed in future SEER releases or through registry-level analyses, the post-2020 incidence estimates should be considered potentially influenced by ascertainment changes. Additional limitations are inherent to SEER-based observational research. SEER does not provide detailed information on recurrence, resection margins, chemotherapy, autoimmune disease, performance status, or comorbidity burden, and treatment variables are limited to first-course therapy [6,10-12]. Treatment allocation is also non-randomized, which limits causal interpretation, particularly for surgery and radiation therapy. This limitation is especially important for radiation therapy, because patients selected for radiation likely had higher-risk disease features, such as advanced stage, positive or close margins, incomplete resection, or unresectable tumors, none of which could be fully adjusted for in the SEER dataset [6,9-12]. In addition, follow-up for the most recent diagnosis years is relatively short, requiring caution when interpreting recent survival patterns. Finally, the incidence-rate dataset and case-level survival dataset were derived from closely related, but not perfectly identical, extracts; therefore, incidence and survival analyses should be viewed as complementary rather than strictly one-to-one matched. For these reasons, the findings should be interpreted as descriptive and prognostic rather than causal, especially for treatment-related associations [6,10-12].

Taken together, these findings position the present study as a contemporary population-level benchmark rather than a definitive treatment-effect analysis. The data support continued use of registry-based cohorts to monitor incidence patterns and identify prognostic factors in rare thoracic malignancies, while also underscoring the need for clinically richer datasets that can better account for stage, resection status, systemic therapy, and longitudinal outcomes. Future work should focus on validating the recent incidence increase in later SEER releases and on integrating registry data with more detailed clinical sources to refine risk stratification and treatment evaluation [4,6,10-12].

## Conclusions

Thymoma remained a rare malignancy in the United States, with incidence broadly stable from 2000 through 2020. Although reported incidence increased during 2021-2023, this pattern should be interpreted cautiously and most likely reflects improved case ascertainment in the SEER November 2025 submission rather than a true epidemiologic change, given the stable population denominators, unchanged ICD-O-3 codes, and marked increase in laboratory-only reported cases. Older age, larger tumor size, and type B3 histology were associated with worse survival, while surgery showed the strongest association with improved overall survival, consistent with its established role as the cornerstone of thymoma management. Radiation therapy was not independently associated with survival in the adjusted model; however, this finding should not be interpreted as evidence against radiotherapy efficacy because treatment selection was likely influenced by unmeasured disease severity, including stage, resection completeness, and other high-risk features not captured in SEER. In addition, the absence of Masaoka-Koga staging in SEER prevents stage-adjusted risk stratification, so the reported hazard ratios should be understood as prognostic associations rather than stage-independent effects. Overall, these findings provide an updated national epidemiologic benchmark and highlight the need for future population-based studies using datasets with more detailed clinical information, including stage, resection margin status, recurrence, and systemic therapy data.
